# Chiral HPLC Separation of Phosphonothrixin and Phosphonate Derivatives and Electronic Circular Dichroism Characterization of the Isolated Enantiomers

**DOI:** 10.1002/elps.70128

**Published:** 2026-07-10

**Authors:** Mirna Maalouf, Maximilian Knab, Leon Gessner, Marc Engelhardt, Stella Friedrich, Harald Gross, Frank Boeckler, Chambers C. Hughes, Michael Lämmerhofer

**Affiliations:** ^1^ Institute of Pharmaceutical Sciences Pharmaceutical (Bio‐)analysis University of Tübingen Tübingen Germany; ^2^ Department of Microbial Bioactive Compounds Interfaculty Institute of Microbiology and Infection Medicine (IMIT) University of Tübingen Tübingen Germany; ^3^ Institute of Pharmaceutical Sciences Pharmaceutical Biology University of Tübingen Tübingen Germany; ^4^ Institute of Pharmaceutical Sciences Molecular Design and Pharmaceutical Biophysics University of Tübingen Tübingen Germany; ^5^ Cluster of Excellence EXC 2124: Controlling Microbes to Fight Infection University of Tübingen Tübingen Germany; ^6^ German Center for Infection Research (DZIF), Partner Site Tübingen Tübingen Germany

**Keywords:** chiral stationary phase, electronic circular dichroism, enantioseparation, herbicides, phosphonates

## Abstract

Phosphonothrixin (PPX), a phosphonate natural product known for its potent herbicidal activity, has attracted renewed interest because of the need for alternative herbicides. Its biological activity is confined to the (*S*)‐enantiomer. Although the only reported enantioselective synthesis is complex, labor‐intensive, and low yielding, direct chiral liquid chromatographic separation is likewise challenging because of its high polarity and lack of aromatic moieties, which limit interactions with commonly used chiral stationary phases (CSPs). In addition, phosphonic acid‐containing compounds tend to chelate metal ions and adsorb to metal surfaces, further complicating chromatographic separation by causing pronounced peak tailing. Here, we describe a reliable and reproducible analytical and preparative method for the direct chiral separation of PPX using a zwitterionic sulfohexyl quinidine and quinine carbamate CSPs, as well as two synthetic phosphonate derivatives using a cellulose tris(3,5‐dichlorophenylcarbamate) stationary phase as an alternative approach. The best separation of PPX was achieved with the zwitterionic sulfohexyl quinidine CSP (Rs = 2.4). The separated enantiomers were isolated, and their chemical and stereochemical identities were confirmed by mass spectrometry, optical rotation, and electronic circular dichroism spectroscopy. This approach enabled the successful isolation of enantiomerically pure (*S*)‐(−)‐ and (*R*)‐(+)‐PPX.

## Graphical abstract

Natural compound phosphonothrixin's herbicidal activity resides exclusively in its (*S*)‐enantiomer, yet existing enantioselective syntheses are cumbersome, and direct chiral LC separation is hampered by the compound's high polarity, lack of aromatic groups, and metal chelation–induced peak tailing. By employing a zwitterionic sulfohexyl quinidine carbamate, stationary phase reliable analytical and preparative chiral separations were achieved, enantiomers isolated, and absolute configurations assigned by optical rotation and ECD spectroscopy.

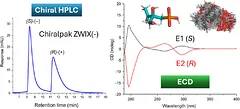

## 1 Introduction

**FIGURE 1:**
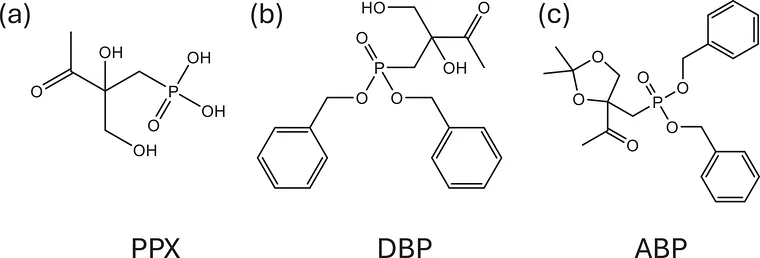
Structure of the tested racemic analytes: (a) phosphonothrixin (PPX), (b) dibenzyl PPX (DBP), and (c) acetonide of dibenzyl PPX (ABP).

Phosphonothrixin (PPX), 2‐hydroxy‐2‐hydroxymethyl‐3‐oxobutylphosphonic acid, is a potent natural herbicide that was first isolated in 1995 from the fermentation broth of *Saccharothrix* sp. ST‐888 (Figure 1a) [1, 2]. Because of this pronounced herbicidal activity, considerable effort has been devoted to accessing the compound by organic synthesis. Several synthetic routes to racemic PPX have been reported [3, 4, 5, 6]. The first enantioselective synthesis, reported in 1997, established the absolute configuration of the natural product as (*S*)‐(−) and at the same time demonstrated that the herbicidal activity is associated with the (*S*)‐enantiomer [7, 8]. In addition, various chemoenzymatic approaches have since been developed to provide access to enantiomerically enriched PPX [9, 10].

For development into a viable herbicide, either high‐yield fermentation or an efficient and scalable synthetic route would be highly advantageous. However, the compound's high‐water solubility and low hydrophobicity result in inefficient extraction from culture and limited retention on conventional reversed‐phase chromatography columns. The enantioselective synthesis of (*S*)‐PPX reported by Nakamura is challenging, low yielding, and of limited scalability, as it relies on demanding transformations such as methyllithium addition, organomagnesium‐mediated deprotonation, low‐temperature steps, and ozonolysis, and affords the product in only 15% overall yield [10]. Racemic synthesis followed by preparative separation of the enantiomers therefore represents an attractive alternative, while also enabling detailed biological characterization of both stereoisomers.

Beyond these synthetic challenges, analytical characterization of PPX is remarkably difficult due to its high‐water solubility and lack of sufficient hydrophobicity hindering retention on classical analytical columns, reversed‐phase, and also chiral stationary phases (CSPs) [11]. Moreover, no derivatization‐based methods, such as Mosher's acid (α‐methoxy‐α‐trifluoromethylphenylacetic acid) analysis or related approaches, have been established for determining PPX enantiopurity. To our knowledge, direct HPLC separation of PPX enantiomers has not yet been reported. This is notable because the preparation of single enantiomers from a synthesized racemate by preparative LC, often referred to as the “racemic approach,” is widely used in chiral drug discovery. When suitable analytical separations are available, they can often be scaled up to preparative quantities and provide access to both enantiomers for biological activity testing [12, 13].

The aim of this work was to establish a reproducible LC method for the chiral separation of PPX or its synthetic phosphonate precursors. To this end, we identified a suitable enantioselective LC method for direct separation of PPX using zwitterionic sulfohexyl quinidine and quinine carbamate CSPs and applied it in preparative HPLC to isolate the pure enantiomers. The configurations of the isolated stereoisomers were assigned using chiroptical methods, including optical rotation measurements, comparison with reported literature values, and electronic circular dichroism (ECD) spectroscopy. Absolute configuration assignments were further supported by comparison of experimental and calculated ECD spectra, which, to our knowledge, have not previously been reported for PPX. The main goal of the study was to achieve direct enantiomer separation of PPX without derivatization, enabling preparative isolation of both enantiomers from a racemic synthesis product of a non‐stereoselective synthesis. In addition, we explored a complementary strategy in which protected phosphonate precursors are separated by preparative chiral HPLC on polysaccharide CSPs, followed by deprotection, to provide access to single enantiomers from racemic starting material.

## 2 Experimental Part

### 2.1 Materials

Racemic PPX (Figure 1a) and dibenzyl PPX (DBP) (Figure 1b) were synthesized as previously described [14]. The synthesis of the acetonide of dibenzyl PPX (ABP) (Figure 1c) is described in the Supporting Information.

Mass‐spectrometry‐grade (Rotisolv, Ultra LC–mass spectrometer [MS]‐grade), methanol (MeOH), 2‐propanol (IPA), and acetonitrile (ACN) were purchased from Carl Roth (Karlsruhe, Germany). Acetonitrile, methanol, and *n*‐heptane, each in HPLC grade, were obtained from Sigma‐Aldrich (Merck, Taufkirchen, Germany). Ultrapure water was produced by additional purification of demineralized water using an ELGA LabWater ultra‐purification system (Celle, Germany). Formic acid (FA), acetic acid (Rotipuran >98%), ammonium acetate, and ammonium formate were obtained from Carl Roth (Karlsruhe, Germany). Ammonia solution (Suprapur quality 25% NH_3_) and diethylamine (DEA) were purchased from Sigma‐Aldrich (Merck).

The chiral columns Chiralpak ZWIX(+) (3.0 mm × 150 mm, 3 µm) (with silica‐bonded zwitterionic (1′*S*,2′*S*)‐*trans*‐2‐sulfohexyl quinine carbamate CSP), Chiralpak IB‐U, and IC‐U (3.0 mm × 100 mm, 1.6 µm) (with immobilized cellulose tris(3,5‐dimethylphenylcarbamate) and cellulose tris(3,5‐dichlorophenylcarbamate), respectively) were obtained from Chiral Technologies (Illkirch‐Graffenstaden, France). RegisCell (4.6 mm × 250 mm, 5 µm) (with cellulose tris(3,5‐dimethylphenylcarbamate) as chiral selector coated on silica) was purchased from Regis Technology (Morton Grove, USA), Kromasil AmyCoat (4.6 mm × 150 mm, 3 µm) (having an amylose tris(3,5‐dimethylphenylcarbamate) chiral selector coated on silica) from Kromasil (AkzoNobel, now Nouryon, Göteborg, Sweden). The prototype column ZWIX(−) (250 mm × 3 mm, 3 µm) (with zwitterionic (1′*R*,2′*R*)‐*trans*‐2‐sulfohexyl quinidine carbamate CSP) was synthesized in‐house [15] and packed by Bischoff Chromatography (Leonberg, Germany).

### 2.2 LC‐UV Measurements

All LC‐UV runs were performed on an Agilent 1260 LC system from Agilent Technologies (Waldbronn, Germany). The system was equipped with a quaternary pump, a degasser, an autosampler, and a UV‐DAD detector. Methanol solutions of all racemic samples were made at a concentration of 1 mg mL^−1^.

### 2.3 LC–MS Measurements

High‐resolution mass‐spectrometry analysis was carried out using an Infinity 1290 series UHPLC system from Agilent Technologies (Waldbronn, Germany) equipped with a binary pump, thermostated column compartment, an HTC‐xt PAL (CTC Analytics AG, Zwingen, CH) autosampler, and a TripleTOF 5600+ MS with a TurboIonSpray Source from SCIEX (Concord, Ontario, Canada).

RPLC conditions were as follows: A Gemini C18, 150 mm × 4.6 mm, 5 µm column was employed for the gradient elution separation. The Mobile phase A consisted of water containing 0.1% (v/v) FA and Mobile phase B of acetonitrile containing 0.1% (v/v) FA. The following gradient profile was used: 5%–100% B in 10 min, 100% B for 2 min, 100%–5% B in 0.1 min, and re‐equilibration for 10 min. The flow rate was 0.5 mL min^−1^. MS was operated in negative ion mode with a cycle time of 600 ms, The source parameters were as follows: curtain gas, 30 psi; source Gas 1, 50 psi; source Gas 2, 40 psi; ionspray voltage floating, 4500 V; ion source temperature, 450°C. Data acquisition used a high‐resolution MRM acquisition mode with a full scan TOF‐MS experiment (250 ms accumulation time) using a mass range of 100–2000 *m*/z followed by two TOF‐MS2 experiments (product ion scans) for the PPX precursor ion [M‐H]^−^ *m*/*z* 197.02 with an accumulation time of 100 ms once with collision energy (CE) of −10 V and declustering potential (DP) −100 V, and the second one with CE −5 V, DP—80 V, with a scan range of 100−2000 *m/z* and 30–500 *m/z*, respectively.

### 2.4 Preparative LC of PPX Enantiomers

PPX enantiomers were isolated by preparative LC using Chiralpak ZWIX(+) (150 mm × 3 mm, 3 µm) and Waters Fraction Collector SF‐3120. The optimal chromatographic conditions employed a mobile phase of MeOH:ACN:H_2_O (49:49:2; v/v/v) containing 100 mM FA and 50 mM NH_3_. The flow rate was 0.2 mL min^−1^, the column temperature was maintained at 15°C, and the detection was performed at 280 nm. A loading study was performed with a 1 mg mL^−1^ sample solution in water. Increasing injection volumes of 30–90 µL were applied to the analytical column to determine the maximum loading that still afforded adequate resolution and reproducible results.

The final conditions for the preparative LC separations were as follows. Racemic PPX was dissolved in water at 1 mg mL^−1^, and 80 µL aliquots were injected. The run time was 20 min under the chromatographic conditions specified above. The two fractions corresponding to the isolated enantiomers, designated *F*1 and *F*2, were collected, whereas an intermediate fraction was discarded to improve enantiomeric purity. The collected fractions were evaporated under a stream of nitrogen, redissolved in 1 mL of water, and subsequently lyophilized using a Labconco FreeZone Freeze Dry System to ensure complete removal of mobile‐phase additives. The purified fractions were then reinjected to confirm successful collection and assess purity, followed by LC–MS analysis. The same conditions were also evaluated on a Chiralpak ZWIX(−) column (3.0 mm × 250 mm, 3 µm).

### 2.5 ECD Measurements

The experimental ECD curves were recorded on a J‐720 Jasco spectropolarimeter with temperature controlled by a PTC‐348WI‐Peltier system in combination with a Julabo F250 recirculation cooler, operated by the Jasco software Spectra Manager version 2. The system was thermostated at 293 K and purged with nitrogen (nitrogen 5.0, purity 99.999%) for 15 min to remove oxygen from the optical system before switching on the light source. Subsequently, the nitrogen flow rate was adjusted to 5 L min^−1^ using a Hornung N_2_ pressure regulator with an integrated flowmeter to minimize absorbance of oxygen in the wavelength range of interest.

Each compound was dissolved in H_2_O (Milli*Q* water) and transferred into a quartz micro cuvette with a 0.2‐cm path length. Spectra were recorded from 185 to 400 nm, at 1.0 nm intervals and a scan speed of 50 nm min^−1^. Three accumulations were averaged for each spectrum. If necessary, the samples were diluted until the Photomultiplier Tube High Tension (PMT HT) voltage, which was monitored in parallel, showed a value below 800 V. The data were saved in the  .jws format, and the spectra were processed with the Spectra Manager version 2 software, background corrected by subtracting the spectrum of the neat solvent recorded under identical conditions, and slightly smoothed.

### 2.6 Measurement of Optical Rotations

The angles of rotation were measured with a Jasco P‐2000 polarimeter, using a cylindrical quartz cell with an inner diameter of 3.5 mm and a path length of 10 mm. The measurement started first by calibrating with the blank (neat solvent), followed by its sample solutions. Each compound was dissolved in H_2_O (Milli*Q* water), transferred into the quartz cell, and ensured that the solution was clear and free from any air bubbles. Ten concurrent readings of the sample were taken at room temperature and at a wavelength of 589 nm of the D‐line of the sodium lamp, and its mean value was considered. The optical rotation data were saved in the  .jws format, and the specific optical rotation was calculated according to the formula ${\left[a\right]}_{\lambda }^{T}$ = *α*/(*c* × *l*) × 10^4^, with *T*, the temperature in °C; *λ*, the wavelength of the light source (here the sodium D‐line at 589 nm); *α*, the rotation angle in degrees; *c* in (g 100 mL^−1^) and *l* in mm.

### 2.7 In Silico Calculation of ECD Spectra

Conformational analysis of (*R*)‐ and (*S*)‐enantiomers of PPX as well as for the mono‐, and di‐anionic phosphonate species was performed using the CREST [16] program suite with the GFN2‐xTB [17] method developed by Grimme et al. For conformations within an energy window of 5 kcal/mol relative to the lowest energy structure, each resulting geometry was subjected to ab initio geometry optimization at the B3LYP‐D3/def2‐TZVP level [18, 19, 20] of theory using TURBOMOLE 7.7.1 [21] on the JUSTUS2‐bwHPC Cluster [22]. For the neutral PPX species, a total of 127 and 134 optimized conformers were obtained for the (*R*)‐ and (*S*)‐enantiomers, respectively. For the mono‐anionic phosphonate species, 113 conformers were identified for the (*R*)‐enantiomer and 123 for the (*S*)‐enantiomer. In the case of the di‐anionic phosphonate species, 26 optimized conformers were obtained for each enantiomer. ECD and ORD spectra were calculated for all optimized conformers of the enantiomers on a B3LYP/def2‐TZVP level of theory using TURBOMOLE. All calculations were done with the COSMO [23] extension to approximate solvent effects in aqueous environments. Spectra were calculated for the first 100 singlet excitations. Each excitation was represented by a Gaussian band shape, with the center of the Gaussian corresponding to the calculated excitation wavelength and the full width controlled by a chosen standard deviation. For each transition, the rotatory strength was converted to molar ellipticity (mdeg) to produce CD spectra, whereas the oscillator strength was transformed into molar absorption coefficients (*ε*) to generate UV/Vis spectra. To account for the conformational ensemble at room temperature, the ground‐state electronic energies of all conformers were extracted and converted to relative energies. From these, Boltzmann weights were computed at 298 K and normalized. The calculated spectra for each conformer were multiplied by the respective Boltzmann weight and subsequently summed across all conformers to yield Boltzmann‐weighted spectra. Protonation state analysis of the two enantiomers at the physiological pH = 7.0 (±1.0) was done using Schrödingers’ LigPrep module with the Epikx tool included [24, 25]. The calculated spectra of the enantiomers corresponding to protonation states present at pH 7 were weighted according to their respective population fractions derived from the protonation state analysis and combined to obtain population‐averaged spectra. Gaussian broadening was then applied using a range of standard deviations (*σ* = 0.2–1.0 eV, in increments of 0.1 eV). Additionally, spectra were generated for each enantiomer and protonation state individually, without applying population weighting or prorating. To accurately compare calculated CD spectra with experimental measurements, calculated UV/Vis absorption spectra were aligned with the experimental HT signal. Because small systematic errors in excitation energies can lead to wavelength shifts, we determined the necessary nm shift by matching the overall shape of the calculated UV/Vis curves to the experimental HT profile. Wavelength adjustment was then applied to the calculated ECD spectra (+46 nm) to ensure that the simulated and experimental features were comparable. Plots of the experimental spectra and the calculated spectra of the mono‐ and di‐anionic phosphonate (*R*)‐ and (*S*)‐variants in a range of 185–350 nm for *σ* = 0.6 eV were generated through custom in‐house Python scripts utilizing the *matplotlib* [26] library.

## 3 Results and Discussion

### 3.1 Column Screening

PPX presents a major challenge for LC enantiomer separation. The compound is hydrophilic and therefore predominantly water soluble, and it lacks an aromatic moiety that could enable favorable directional π–π interactions with complementary binding sites on CSPs. In addition, the hydroxyl groups typically make an unfavorable free energy contribution to analyte‐stationary phases in aqueous eluents [27] and, therefore, contribute little to retention or chiral recognition. The phosphonic acid moiety further complicates chromatographic analysis, as it often causes broad, tailing peaks because of interactions with stainless steel surfaces, slow adsorption–desorption kinetics, and heterogeneous mass transfer [28, 29]. For this reason, both PPX (Figure 1a) as well as the DBP and ABP (Figure 1b,c), which were expected to alleviate some of these limitations, were included in column screening and method development, respectively, to have an alternative strategy of preparative isolation via the phosphonate route if the direct enantiomer separation of PPX would have failed.

#### 3.1.1 Phosphonates on Polysaccharide CSPs

In our study, four columns based on different polysaccharide‐type CSPs with different carbohydrate backbone (amylose and cellulose) and different carbamate derivatives (3,5‐dimethylphenyl and 3,5‐dichlorophenyl) were initially tested. These two classes of polysaccharide type CSPs were selected for their wide spectrum of applicability (polysaccharide CSPs). Although both PPX and DBP were tested initially, only DBP enantiomers could be separated on the cellulose‐based columns.

**FIGURE 2:**
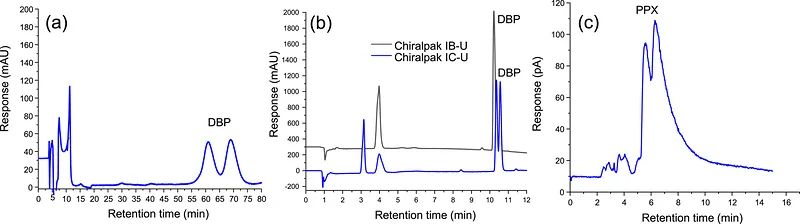
Screening results of (a) dibenzyl PPX (DBP) on a RegisCell column (250 mm × 4.6 mm, 5 µm) using normal phase (NP) mode, (b) DBP on two immobilized cellulose‐based sub‐2 µm UHPLC columns (100 mm × 3 mm, 1.6 µm) Chiralpak IB‐U (black) and IC‐U (blue), and (c) phosphonothrixin (PPX) on a ZWIX(+) column (150 mm × 3 mm, 3 µm).

First, the enantioselective separation of DBP was tested on the polysaccharide‐type CSPs in NP and RP mode. The initial screening of NP mode was performed on cellulose tris(3,5‐dimethylphenylcarbamate) (RegisCell) and amylose tris(3,5‐dimethylphenylcarbamate) (AmyCoat) columns using IPA/heptane (5:95, 10:90, and 20:80; v/v) containing 0.1% (v/v) TFA (v/v) at 10°C and flow rate of 1 mL min^−1^. Amylose‐based polysaccharide AmyCoat demonstrated a considerably weaker capacity to recognize enantiomers of DBP than the cellulose‐based RegisCell. Of the two tested compounds, only DBP showed enantiomer separation on the RegisCell column. Figure 2a depicts the enantiomer separation of DBP on RegisCell column with NP eluent. The two enantiomers are close to baseline separated; however, the run times were extremely long with only 5% (v/v) polar modifier IPA. Higher IPA content led to a significant decrease in retention, but unfortunately also in resolution (Table 1). Although NP conditions with 5% (v/v) IPA appeared adequate for effective preparative LC enantiomer separation, further optimization was pursued to enhance resolution, recovery, and process robustness.

**TABLE 1:** Chromatographic parameters for dibenzyl phosphonothrixin (PPX) (DBP) under different experimental conditions.

Tested conditions	Retention factor *k* ^a^		Separation factor α^b^	Resolution Rs	Column
	Peak 1	Peak 2			
Isopropanol/Heptane (5:95, v/v) + 0.1% (v/v) TFA	20.26	22.95	1.13	1.24	RegisCell
Isopropanol/Heptane (10:90, v/v) + 0.1% (v/v) TFA	9.20	9.71	1.06	0.47	RegisCell
Isopropanol/Heptane (20:80, v/v) + 0.1% (v/v) TFA	2.98	3.37	1.13	0.93	RegisCell
Mobile phase A: H_2_O + 0.1% (v/v) TFA; B: MeOH + 0.1% (v/v) TFA; Gradient: 10% B to 80% B in 30 min, 25°C	*tR*_1_:23 min	—	1.0	0.0	IC‐U
Mobile phase A: H2O + 0.1% (v/v) TFA; B: ACN + 0.1% (v/v) TFA; Gradient: 10%–80% B in 30 min, 25°C	10.35	10.56	1.02	1.09	IC‐U
Mobile phase A: H_2_O + 0.1% (v/v) TFA; B: ACN + 0.1% (v/v) TFA; Gradient: 20% B to 80% B in 30 min, 15°C	8.48	8.81	1.04	1.19	IC‐U
Mobile phase A: H2O + 0.1% (v/v) TFA; B: ACN + 0.1% (v/v) TFA; Gradient: 10% B to 80% B in 30 min, 8°C	10.96	11.21	1.02	1.05	IC‐U
Mobile phase A: H_2_O + 0.1% (v/v) TFA; B: ACN + 0.1% (v/v) TFA; Gradient: 20% B to 40% B in 30 min, 15°C	10.74	11.26	1.04	1.17	IC‐U
Mobile phase A: H2O + 0.1% (v/v) TFA; B: ACN + 0.1% (v/v) TFA at 30% B, 15°C	4.63	4.94	1.08	1.33	IC‐U

^a^ *t*_0_:2.82 min (RegisCell 250 mm × 4.6 mm; flow rate: 1 mL min−^1^).

^b^Alpha = (*t_R_*_2_ − *t*_0_)/(*t_R_*_1_ − *t*_0_).

In addition to these NP tests with coated polysaccharide CSPs, also two immobilized cellulose‐based sub‐2 µm UHPLC columns, Chiralpak IB‐U cellulose tris(3,5‐dimethylphenylcarbamate) chiral selector and IC‐U with cellulose tris(3,5‐dichlorophenylcarbamate) chiral selector, were screened in the RP mode using a gradient run with MeOH and ACN, respectively, as organic modifier at column temperature of 25°C and flow rate of 0.5 mL min^−1^. The results for DBP are shown in Figure 2b. Only the IC‐U column with the cellulose tris(3,5‐dichlorophenylcarbamate) chiral selector exhibited enantioselectivity. These findings indicate that the substituents on the phenylcarbamate moiety of cellulose strongly influence enantiorecognition [30, 31, 32]. In the case of IC‐U, the electron‐withdrawing substituents reduce the electron density of the aromatic ring and increase the acidity of the NH proton, which appears to favor interactions between selector and analyte. This effect is likely mediated by improved π–π interactions between the phenyl rings of the selector and the benzyl groups of the analyte, possibly supported by hydrogen bonding and dipole–dipole interactions.

#### 3.1.2 PPX on Zwitterionic Quinine Carbamate CSP

Screening experiments for PPX were unsuccessful under the NP and RP conditions described above, with no indication of enantiomer separation. The highly polar nature of PPX, its lack of aromatic ring systems, the strong solvation of its two hydroxy groups in aqueous eluents, which limits their interaction with the CSP, and the absence of extended bulky hydrophobic substituents all contributed to the poor retention observed on polysaccharide CSPs under RP conditions. Thus, enantioseparation of underivatized PPX proved to be highly challenging. Thus, two columns based on quinine and quinidine carbamate‐based CSPs were evaluated having different carbamate residues ((1*S*,2*S*)‐ or (1*R*,2*R*)‐*trans*‐2‐aminocyclohexanesulfonic acid). They were selected because of their dedicated scope for chiral acidic compounds, that is, free PPX.

Among all tested columns, only the ZWIX(+) column exhibited enantioselectivity and enabled separation of PPX enantiomers, as shown in Figure 2c. This separation can be attributed to the protonated quinuclidinium moiety, which acts as a weak anion exchanger, whereas the (1*S*,2*S*)‐*trans*‐2‐aminocyclohexanesulfonic acid moiety serves as an intramolecular counterion that moderates the otherwise strong interaction of the phosphonic acid analyte with the WAX site. The initial screening conditions consisted of ACN/MeOH/H_2_O (49:49:2, v/v/v) containing 25 mM FA and 12.5 mM DEA, at a flow rate of 0.3 mL min^−1^ and a column temperature of 15°C. Because the compound lacks a strong chromophore for sensitive UV detection, a charged aerosol detector (CAD) was used. Sequential optimization of the parameters affecting enantioseparation was then performed and is discussed in detail in the following section.

### 3.2 Fine Tuning of Chiral LC Separation Conditions

In the second step, chromatographic conditions for DBP on IC‐U and for PPX on ZWIX(+) were optimized by systematically evaluating the effects of organic modifier, flow rate, temperature, buffer type, and concentration.

#### 3.2.1 Dibenzyl Phosphonate Derivatives of PPX (DBP and ABP) on Chiralpak IC‐U

**FIGURE 3:**
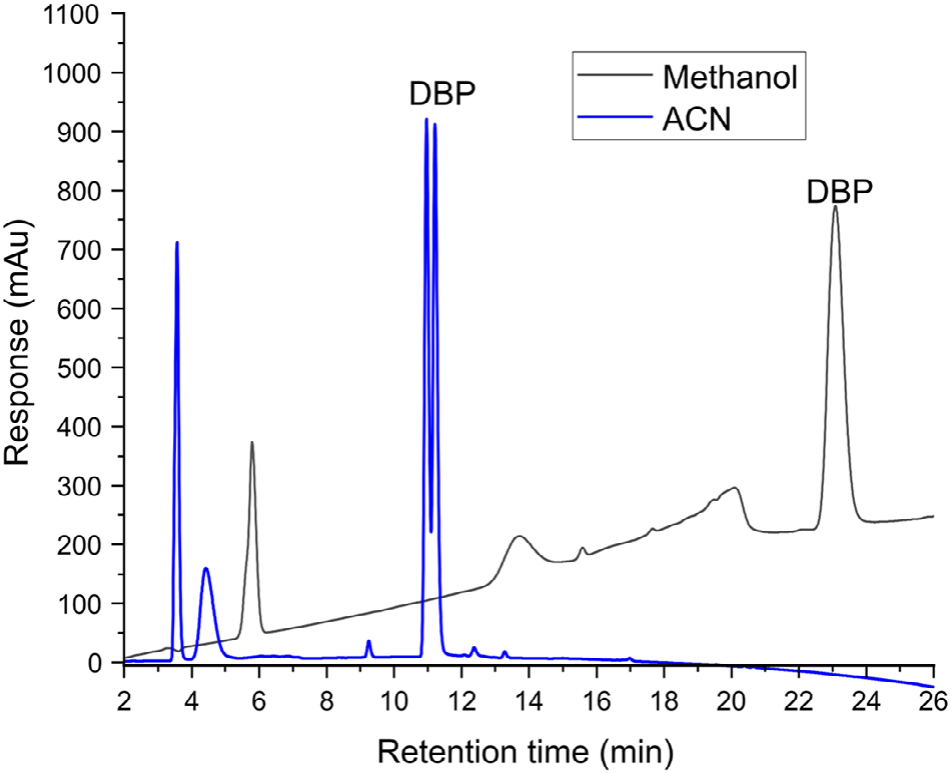
Effect of organic modifier methanol (shown in black) versus acetonitrile (shown in blue) on the enantioseparation of dibenzyl PPX (DBP) on a Chiralpak IC‐U column (100 mm × 3 mm, 1.6 µm).

The column screening indicated a reasonable enantiomer separation capacity of the IC‐U column for DBP in RP elution mode. Yet, further optimization of the experimental conditions was required to achieve full baseline separation. The type of organic modifier typically has a profound influence on enantiomer selectivity and efficiency on polysaccharide CSPs. Hence, MeOH and ACN were compared with gradient elution from 10% to 80% organic modifier and 0.1% (v/v) TFA in water. The substitution of ACN by MeOH led to a loss of enantioselectivity for DBP as well as to a significant decrease in the column efficiency in terms of plate number and peak shape (Figure 3). The plate number decreased by a factor of 3 for MeOH relative to ACN. These effects can be attributed to the π–π interaction between the benzyl ring of the analyte and the aromatic moiety of the selector that can be balanced more efficiently by ACN. Like in RPLC, ACN can disrupt hydrophobic interactions more efficiently and has higher elution strength also in RP‐type chiral separation on polysaccharide phases. Moreover, due to its lower viscosity, ACN has favorable mass transfer properties compared to MeOH and gives therefore narrower peaks. In the next step, the effect of TFA (used initially in the screening because the free phosphonic acid PPX was also tested) was investigated, which apparently had no positive effect on the enantioseparation of DBP and was, therefore, omitted from the mobile phase in further experiments. Finally, isocratic conditions were adjusted, and 30% B (using ACN as organic modifier) showed the best resolution and a relatively fast separation.

Temperature is a parameter with influence on thermodynamics and kinetics of the separation. It is therefore a prime variable for evaluation and optimization yet may have an opposing effect on enantioselectivity and efficiency, that is, typically higher enantioselectivity at lower temperatures and higher efficiencies at elevated temperatures. Hence, the column temperature was tested at 8°C and 25°C under same conditions. The resolution increased from 8°C to 25°C.

**FIGURE 4:**
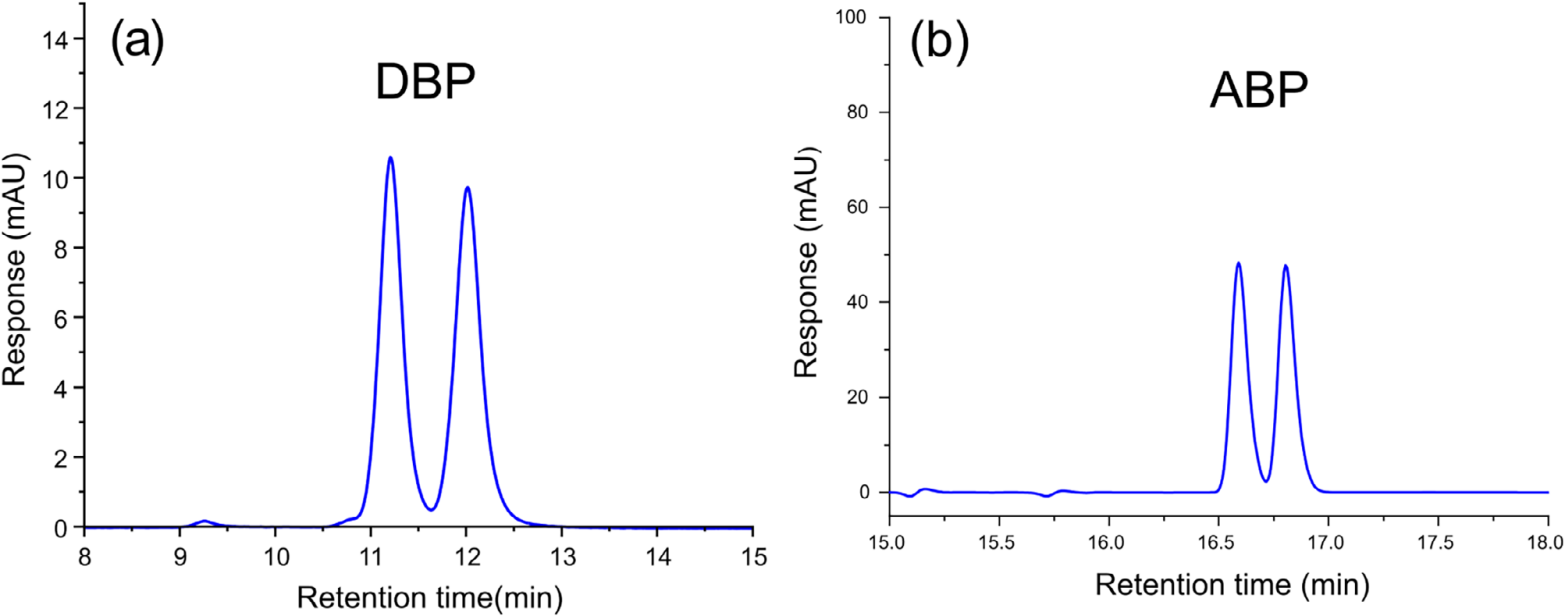
Optimized separation conditions of (a) dibenzyl PPX (DBP) and (b) acetonide of dibenzyl PPX (ABP) on a Chiralpak IC‐U column (100 mm × 3 mm, 1.6 µm). Experimental conditions: (a) Mobile phase A: H_2_O; Mobile phase B: ACN; isocratic elution: 30% (v/v) B, flow rate: 0.2 mL min^−1^, column temperature: 15°C, (b) Mobile phase A: H_2_O; Mobile phase B: ACN; gradient elution from 10% to 100% (v/v) B in 20 min, flow rate: 0.2 mL min^−1^, column temperature: 15°C.

A van Deemter curve was then recorded under the chosen conditions testing the effect of six different flow rates on the column efficiency to find the minimum that gives the best separation (Figure S1). The highest plate number and best resolution were obtained at a linear flow velocity of 0.35 mm/s which corresponds to a flow rate of 0.1 mL min^−1^. As these conditions are slow, a factor 2 higher flow rate was employed (0.2 mL min^−1^) as a compromise while maintaining almost a baseline separation of the DBP enantiomers. The final chosen conditions were as follows: Mobile phase A: H_2_O; Mobile phase B: ACN; isocratic elution: 30% B, flow rate: 0.2 mL min^−1^, column temperature: 15°C, detection wavelength: 260 nm, sample concentration: 1 mg mL^−1^, and injection volume: 2 µL (Figure 4a).

Another derivative, the ABP (Figure 1c) was also evaluated. Using the IC‐U column (100 × 3, 1.6 µm) and a mobile phase composed of H_2_O (A) and ACN (B) with gradient elution from 10% to 100% B in 20 min and a flow rate of 0.2 mL min^−1^ at a column temperature of 15°C a separation of the enantiomers with Rs 1.48 resulted (see Figure 4b).

After transfer to a column with larger particles, for example, Chiralpak IC‐3 (3 µm particles of the same CSP), and compensating for the loss in efficiency by use of a longer column (250 mm length), these methods would be suitable for preparative LC separation of the two PPX derivatives, DBP and ABP. However, because PPX showed a positive screening result on ZWIX(+), we next explored its direct preparative LC separation on this column.

#### 3.2.2 Phosphonic Acid PPX on Chiralpak ZWIX(+)

The separation of the PPX on the Chiralpak ZWIX(+) phase follows an enantioselective anion‐exchange process. Phosphonic acids give a very strong ion‐exchange interaction on the quinuclidinium moiety of the chiral ZWIX selector. The sulfonic acid moiety of the ZWIX(+) selector weakens this interaction by an intramolecular counterion effect. For efficient chiral separations, these interactions need to be finely tuned to result in enantiomer separation. In the case of PPX, first the organic modifier effect (MeOH, ACN, and MeOH/ACN mixture) was tested in isocratic elution mode. The following mobile phases were prepared: MeOH:H_2_O (98:2; v/v), ACN:H_2_O (98:2; v/v), and ACN:MeOH:H_2_O (49:49:2; v/v) always containing 100 mM FA and 50 mM aqueous NH_3_. It turned out that the mixture of MeOH/ACN showed better selectivity than MeOH alone and better peak shape than ACN (Table 2).

**TABLE 2:** Chromatographic parameters for phosphonothrixin (PPX) on ZWIX(+) under different experimental conditions

Tested conditions	Retention factor *k*		Separation factor *α*	Resolution Rs
	Peak 1	Peak 2		
MeOH:H_2_O (98:2; v/v) + 100 mM FA + 50 mM NH_3_	1.71	2.21	1.30	1.18
ACN:H_2_O (98:2; v/v) + 100 mM FA + 50 mM NH_3_	2.01	2.44	1.21	0.59
MeOH:ACN:H_2_O (49:49:2; v/v/v) + 100 mM FA + 50 mM NH_3_	1.99	2.42	1.22	1.18
MeOH:ACN:H_2_O (49.5:49.5:1; v/v/v) + 100 mM FA + 50 mM NH_3_	2.23	2.75	1.23	0.70
MeOH:ACN:H_2_O (48:48:4; v/v/v) + 100 mM FA + 50 mM NH_3_	1.51	1.92	1.27	1.03
MeOH:ACN:H_2_O (49:49:2; v/v/v) + 50 mM FA + 50 mM NH_3_	2.01	2.44	1.21	0.59
MeOH:ACN:H_2_O (49:49:2; v/v/v) + 100 mM FA + 50 mM NH_3_	1.99	2.42	1.22	1.18
MeOH:ACN:H_2_O (49:49:2; v/v/v) + 150 mM FA + 50 mM NH_3_	1.84	2.34	1.27	0.64
MeOH:ACN:H_2_O (49:49:2; v/v/v) + 50 mM FA + 100 mM NH_3_	1.46	1.75	1.20	0.49
MeOH:ACN:H_2_O (49:49:2; v/v/v) + 200 mM FA + 100 mM NH_3_	1.41	1.76	1.25	0.70

Next, the effect of water content was investigated by using 1%, 2%, and 4% (v/v) H_2_O in the mobile phase, whereby 2% (v/v) water content showed a better enantioseparation (Rs 1.1) in comparison to 1% (v/v) water (Rs 0.7) followed by a slight decrease in resolution in the case of 4% (v/v) water content (Rs 1.0) (Table 2).

**FIGURE 5:**
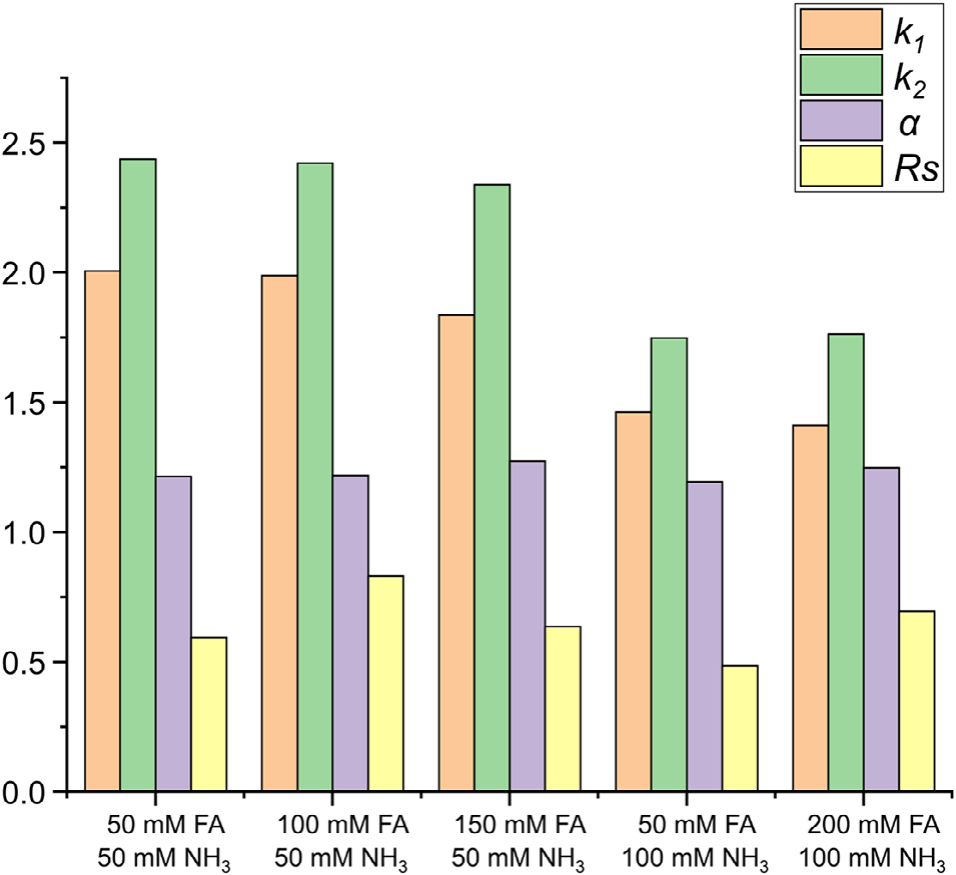
Effect of the buffer concentration as well as the acid‐to‐base ratio on the enantioseparation of phosphonothrixin (PPX) expressed in terms of retention factor, separation factor, and resolution. FA, formic acid.

Last, the effect of the buffer concentration as well as the acid‐to‐base ratio was tested and optimized (Figure 5). The resolution was the largest at a molar acid‐to‐base ratio (FA to NH_3_) of 2:1. The higher their concentrations, the lower the retention factors, and no gain in Rs was found when the FA:NH_3_ ratio was increased from 100/50 to 200/100 mM (Table 2, Figure 5).

The final chosen conditions were as follows: The mobile phase was composed of MeOH:ACN:H_2_O (49:49:2; v/v/v) containing 100 mM FA and 50 mM NH_3_. The flow rate was 0.2 mL min^−1^ at a column temperature of 15°C, and detection wavelength of 280 nm. A sample concentration of 1 mg mL^−1^ and an injection volume of 4 µL was selected for this experiment (Table 2).

### 3.3 Preparative LC Enantiomer Separation of PPX

On the basis of the results described above, underivatized PPX was selected for the preparative isolation of the individual enantiomers. Preparative LC enantioseparation provides a straightforward approach to accessing both enantiomers from a synthetic racemate of a chiral compound. Separation conditions can be scaled linearly to the corresponding column dimensions, which may be selected according to the amount of substance required. In the present study, an analytical‐scale column was used to obtain milligram quantities of each enantiomer. A critical factor in preparative LC is the loading capacity of the column, which must be determined experimentally. Under overload conditions, resolution decreases, which negatively affects the enantiomeric purity that can be achieved. In general, a compromise exists between yield and enantiomeric purity, as higher sample loads typically increase yield at the expense of enantiomeric purity.

**FIGURE 6:**
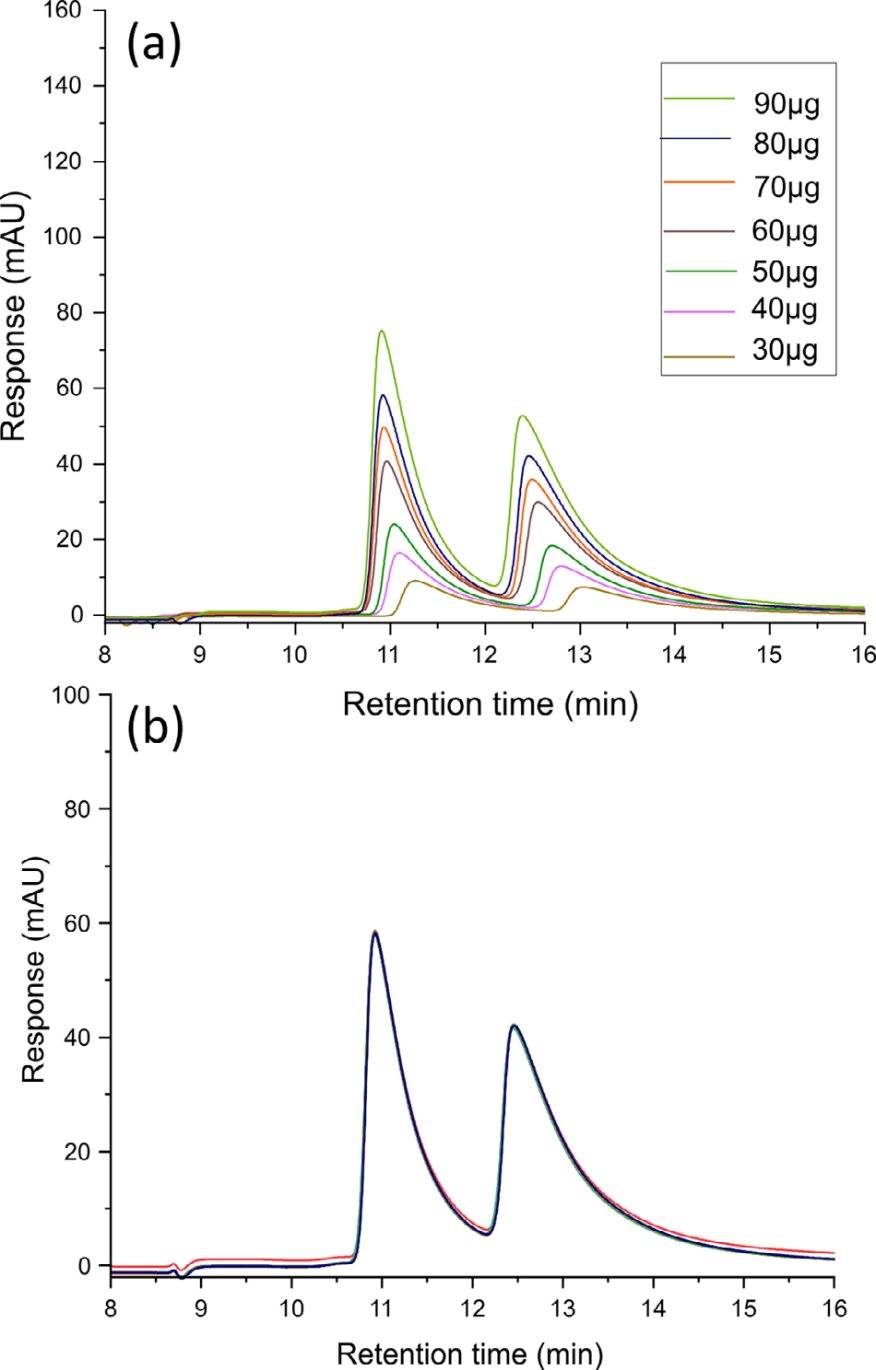
(a) Sample loading study for preparative separation of phosphonothrixin (PPX) on a ZWIX(+) column and (b) repeatability (n=15) of the optimized preparative separation using 80 µg injections.

Hence, the sample loading capacity was investigated using a 1 mg mL^−1^ sample. As can be seen in Figure 6a; by increasing the injection volume, efficient enantiomer separation of the PXP compound was achieved with a resolution of 1.58 up to 80 µL corresponding to an 80 µg sample load on the 150 mm × 3 mm ID ZWIX(+) column. However, further increasing the injection volume led to a decrease in resolution. Therefore, 80 µL was chosen for the collection of individual enantiomers. In order to assess whether an automated collection based on retention times with a fraction collector is possible, the repeatability of the optimized preparative separation at 80 µL injection was tested to guarantee an effective separation of the enantiomers. The result of 15 chromatographic runs was evaluated and is shown in Figure 6b. A high repeatability was observed, making the separation suitable for automated fractionation (RSD of *t*_R_ of first and second eluted enantiomer peaks were 0.024% and 0.023%, respectively). Due to the tailing, a middle fraction was removed and reinjected to guarantee a high enantiomeric purity at the expense of yield.

The collected pooled fractions, designated as *F*1 and *F*2, were evaporated to dryness and reinjected onto a Chiralpak ZWIX(+) column (150 mm × 3 mm, 3 µm) to determine their purity. As shown in Figure S2, Fraction 1 (black) contained a single enantiomer (E1) with an estimated enantiomeric purity of >99%, whereas Fraction 2 (red) showed significant enantiomeric impurity, with enantiomer E2 exhibiting an estimated purity of approximately 94.5%. Purity values were determined from peak areas using tangential integration and peak splitting at the shoulder, respectively.

Finally, the chemical identity of the collected fractions was confirmed by RPLC–ESI–QTOF–MS on a Gemini C18 column (150 mm × 4.6 mm, 5 µm), as shown in Figure S3. The stereochemical identity of the isolated enantiomers is discussed below. Thus, the present preparative LC method using Chiralpak ZWIX(+) provides an attractive route to access the individual enantiomers of both optical antipodes.

### 3.4 Evaluation of ZWIX(−) for Reversal of the Elution Order

**FIGURE 7:**
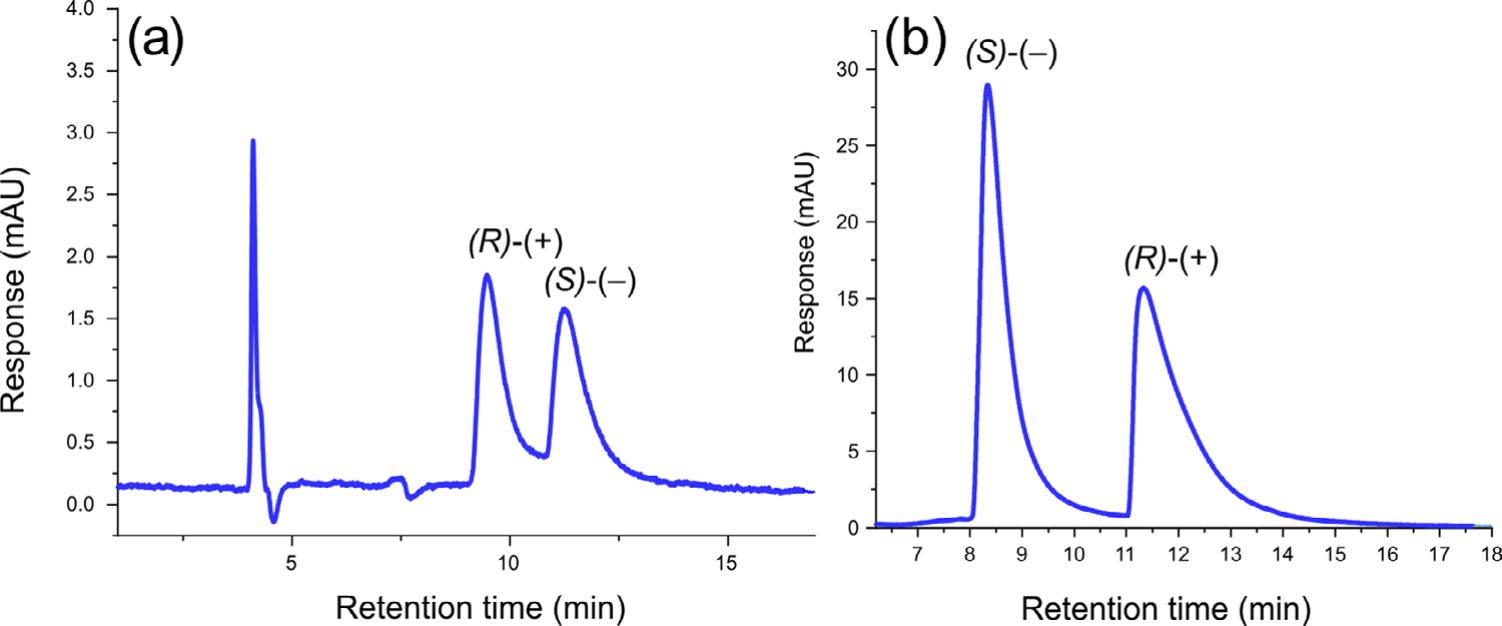
Optimized separation of phosphonothrixin (PPX) on (a) a ZWIX(+) column (150 mm × 3 mm, 3 µm) and (b) a ZWIX(–) column (250 mm × 3 mm, 3 µm).

To investigate whether the elution order on ZWIX(−) (quinidine‐based) is reversed relative to ZWIX(+) (quinine‐based) because of their pseudo‐enantiomeric relationship (due to opposite configurations at carbon 8 and 9), a 10 µL aliquot of a 1 mg mL^−1^ PPX solution was injected onto a ZWIX(−) column (250 mm × 3 mm, 3 µm) under the same chromatographic conditions, using a flow rate of 0.4 mL min^−1^. The isolated E1 and E2 fractions obtained from ZWIX(+) (Figure 7a) were then reinjected onto ZWIX(−). As expected, the elution order was reversed, and full baseline separation was achieved on the ZWIX(−) column (Rs = 2.4) (Figure 7b and Figure S6). Both peaks were subsequently collected preparatively under the same chromatographic conditions using an injection volume of 80 µL and a fraction collector (Figure S4). The pooled fractions were worked up according to the same procedure used for separations on ZWIX(+). Reinjection of the isolated enantiomers onto the ZWIX(−) column indicated high enantiomeric purity, with no detectable enantiomeric impurities (Figure S5). These results show that the higher resolution obtained on ZWIX(−) enables isolation of the enantiomers with improved purity.

Finally, the configurations of the two isolated enantiomers were assigned by optical rotation measurements. According to the literature, the natural enantiomer, (*S*)‐(−)‐PPX, isolated from *Saccharothrix* sp. ST‐888, has a specific rotation of ${\left[\alpha \right]}_{D}^{25}$ = –4.1 [33]. The first‐eluting peak on Chiralpak ZWIX(−) (*t*_R_ = 8.3 min) showed a specific rotation of ${\left[\alpha \right]}_{D}^{25}$ = −4.4) (*c* = 1.00, H_2_O) and was, therefore, identified as (*S*)‐(−)‐PPX. The second‐eluting peak (*t*_R_ = 11.2 min) showed a specific rotation of ${\left[\alpha \right]}_{D}^{25}$= +3.3 (*c* = 1.00, H_2_O) and was accordingly assigned as (*R*)‐(+)‐PPX. Both isolated enantiomers were obtained with an ee >99% (Figure S5).

### 3.5 ECD Spectra of PPX Enantiomers

**FIGURE 8:**
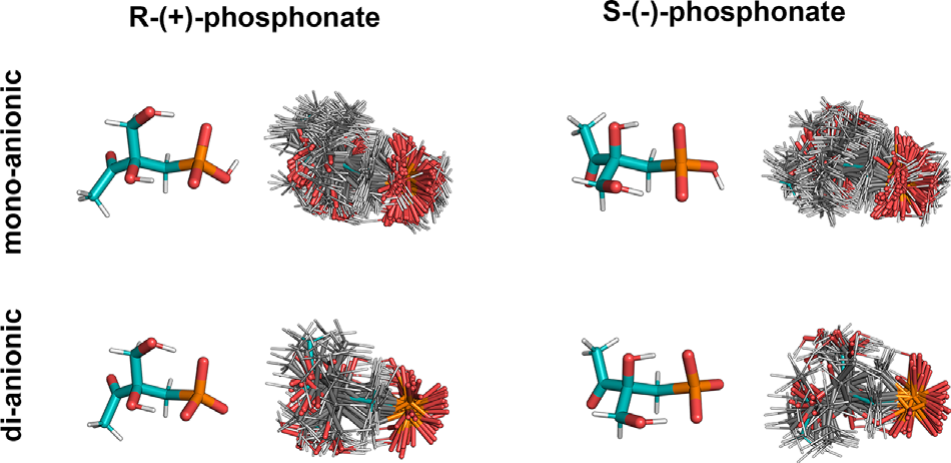
Lowest energy conformation of the mono‐ and di‐anionic phosphonate species of (*R*)‐(+)‐ and (*S*)‐(–)‐phosphonothrixin (PPX). The single molecule represents the respective lowest energy conformation, whereas the ensemble shows all superposed conformations of the corresponding species. Figures were prepared using PyMOL [37].

**FIGURE 9:**
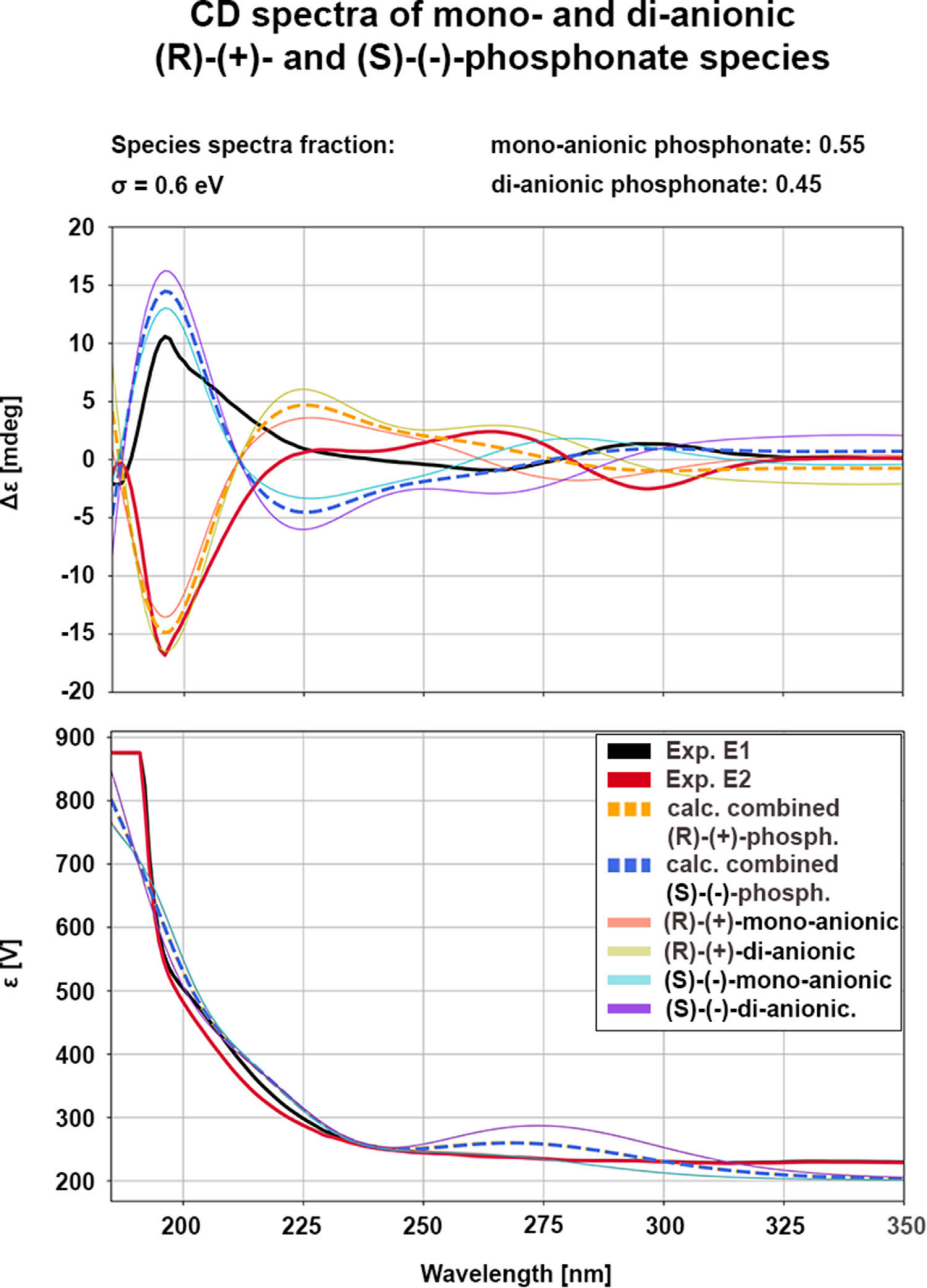
Experimental and population‐averaged (55%/45%), calculated ECD and UV/Vis spectra of mono‐ and di‐anionic phosphonate species of (*R*)‐(+)‐ and (*S*)‐(–)‐enantiomers of phosphonothrixin (PPX). Thin lines represent the spectra of mono‐ and di‐anionic species separately. Dotted lines represent the resulting prorated, combined spectra of the respective enantiomer. E1 and E2 refer to the first (E1) and second (E2) eluted enantiomers on a Chiralpak ZWIX(−) column.

ECD spectroscopy is an essential analytical technique used to characterize chiral molecules through their distinct absorption of left‐ and right‐handed circularly polarized light. To further examine the enantiomer assignment, the experimental ECD spectra (Figure S7) were compared with calculated ECD spectra for both enantiomers collected from the Chiralpak ZWIX(−) column. For this purpose, a conformational analysis was performed, and the different low energy conformers considered for the ECD calculations (Figure 8). Figure 9 shows the experimental ECD (a) and UV/Vis spectra (b) together with the corresponding calculated population‐averaged spectra for the (*R*)‐ and (*S*)‐enantiomers of the mono‐ and di‐anionic phosphonate species for a broadening factor of *σ* = 0.6 eV. Protonation state analysis indicated a population distribution of 55% *mono*‐anionic and 45% *di*‐anionic species under the investigated conditions. Additionally, alternative population ratios of 60/40 (%) and 50/50 (%) were evaluated to assess the sensitivity of the simulated spectra to variations in protonation state distribution (Figure S8). Although exact agreement in band positions and intensities between calculated and experimental spectra is not expected, the calculated spectra reproduce the overall shape and sign pattern of the main Cotton effects observed experimentally. In particular, the relative sequence and curvature of the dominant spectral features show good qualitative agreement in the overall spectral profile after alignment of the calculated spectra with the experimental UV/Vis profile.

For the experimental E1 spectrum (first eluted enantiomer collected on the ZWIX(−) column), a strong positive Cotton effect in the ∼190–210 nm region followed by a weak negative/near‐zero region at higher wavelengths is well captured by the calculated spectrum of the (*S*)‐enantiomer. In contrast, the experimental E2 spectrum (second eluted enantiomer on the ZWIX(−) column), characterized by an intense negative Cotton effect in the same region and a subsequent positive feature at longer wavelengths, is consistently reproduced by the calculated spectrum of the (*R*)‐enantiomer. Taken together, the agreement in sign, sequence, and overall spectral profile provides strong support for assigning E1 on ZWIX(−) to the (*S*)‐(−)‐enantiomer and E2 (on ZWIX(−)) to the (*R*)‐(+)‐enantiomer.

ECD spectra can be highly sensitive to subtle electronic and conformational effects as well as to the protonation state of functional groups [34, 35, 36]. Although solvent effects were included through a COSMO continuum model to approximate aqueous conditions, explicit solvent interactions and hydrogen‐bonding networks cannot be fully captured by such models. In addition, small variations in conformational populations, potentially influenced by solvent interactions or protonation equilibria, can significantly affect the sign and magnitude of ECD bands. These factors may contribute to deviations between calculated spectra for individual molecular models and experimentally measured spectra. Variations of including the conformational space to a smaller or larger extent and of considering different protonation states were conducted in this study. Thus, because the experimental CD spectra were evaluated in combination with QM‐calculated ECD spectra that reproduce the observed spectral features, the present analysis provides an additional and potentially more direct basis for assessing the configurational assignment of the two enantiomers.

## 4 Conclusion

Renewed interest in the natural product PPX has also brought new synthetic access strategies into focus, including enantioselective synthesis, enzymatic production, and racemic synthesis followed by enantiomer separation. In early‐stage development, the latter approach is particularly attractive, as both enantiomers are typically required and can, in principle, be accessed efficiently by LC separation of racemates. To this end, a liquid chromatographic method for enantiomer separation of PPX was developed that is suitable both for analytical quality control and for preparative isolation of the individual enantiomers. The free phosphonic acid is a particularly challenging analyte because of its high polarity, lack of aromatic moieties, and the tendency of the phosphonic acid group to undergo metal adsorption and complexation, leading to peak tailing. For this reason, two PPX derivatives, namely, DBP and the corresponding acetonide, were also considered for preparative chiral separation. A cellulose tris(3,5‐dichlorophenylcarbamate) CSP enabled baseline separation of the enantiomers of both dibenzyl phosphonate derivatives. Finally, preparative LC enantioseparation was achieved for the free phosphonic acid on a Chiralpak ZWIX column, which provided baseline separation of the PPX enantiomers. The best separation of PPX was achieved with the zwitterionic sulfohexyl quinidine CSP (Rs = 2.4) in about 16 min. Future work might attempt reducing the peak tailing by use of stronger counterions, such as phosphate buffer, in combination with elevated temperatures. The quinidine‐based ZWIX(−) column showed higher resolution than the quinine‐based ZWIX(+) column, in part because of the longer column length, and consequently enabled isolation with higher enantiomeric purity. The isolated enantiomers were characterized by optical rotation and ECD spectroscopy, confirming the successful isolation of enantiomerically pure (*S*)‐(–)‐ and (*R*)‐(+)‐PPX. Overall, this work establishes a successful strategy for accessing PPX enantiomers in milligram quantities, with the potential to be scaled to larger batch sizes.

## Author Contributions

**Mirna Maalouf**: investigation, methodology, data curation, visualization, writing – original draft, writing – review and editing. **Maximilian Knab**: investigation, writing – review and editing. **Leon Gessner**: investigation, writing – review and editing. **Marc Engelhardt**: investigation, writing – review and editing. **Stella Friedrich**: investigation, writing – review and editing. **Harald Gross**: supervision, resources, writing – review and editing. **Frank Boeckler**: supervision, resources, writing – review and editing. **Chambers C. Hughes**: conceptualization, supervision, resources, writing – review and editing. **Michael Lämmerhofer**: conceptualization, methodology, supervision, resources, writing – review and editing.

## Conflicts of Interest

The authors declare no conflicts of interest.

## Supporting information




**Supporting File**: elps70128‐sup‐0001‐SuppMat.docx.

## Data Availability

The data that supports the findings of this study are available in the Supporting Information of this article.
