# Supplementary material for: Chiral HPLC Separation of Phosphonothrixin and Phosphonate Derivatives and Electronic Circular Dichroism Characterization of the Isolated Enantiomers
Source: Electrophoresis. 2026 Jul 10;47(9):853–65. doi: 10.1002/elps.70128 (PMC13572893; doi:10.1002/elps.70128)
Supplement: Supplementary file 1 [file ELPS-47--s001.docx]

# Supplementary Material

**Chiral HPLC Separation of Phosphonothrixin and Phosphonate Derivatives and ECD Characterization of the Isolated Enantiomers**

Mirna Maalouf ^1^, Maximilian Knab^2^, Leon Gessner^3^, Marc Engelhardt^4^, Enrico Friedrich^1^, Harald Gross^3,5,6^, Frank Böckler^4^, Chambers C. Hughes^2,5,6^*, Michael Lämmerhofer ^1^*

^1^ Institute of Pharmaceutical Sciences, Pharmaceutical (Bio-)Analysis, University of Tübingen, Auf der Morgenstelle 8, 72076 Tübingen, Germany

^2^ Department of Microbial Bioactive Compounds, Interfaculty Institute of Microbiology and Infection Medicine (IMIT), University of Tübingen, 72076 Tübingen, Germany

^3^ Institute of Pharmaceutical Sciences, Pharmaceutical Biology, University of Tübingen, Auf der Morgenstelle 8, 72076 Tübingen, Germany

^4^ Institute of Pharmaceutical Sciences, Molecular Design and Pharmaceutical Biophysics, University of Tübingen, Auf der Morgenstelle 8, 72076 Tübingen, Germany

^5^ Cluster of Excellence EXC 2124: Controlling Microbes to Fight Infection, University of Tübingen, 72076 Tübingen, Germany

^6^ German Center for Infection Research (DZIF), Partner Site Tübingen, 72076 Tübingen, Germany

* Author for correspondence:

E-mail: chambers.hughes@uni-tuebingen.de

E-mail: [michael.laemmerhofer@uni-tuebingen.de](mailto:michael.laemmerhofer@uni-tuebingen.de)

ORCID: Michael Lämmerhofer: 0000-0002-1318-0974

0.0

0.5

1.0

1.5

2.0

2.5

8

10

12

14

16

18

20

22

24

26

H [µm]

u [mm/s]

Fig. S1: Van Deemter curve of DBP recorded on Chiralpak IC-U column using 6 different flow rates to find the minimum which gives the best separation where the red points represent the second eluting peak of DBP, and the black squares represents the first eluting peak of DBP.

Table S 1 Van Deemter terms calculations derived from H/u curve of DBP on Chiralpak ICU

| Model | Van Deemeter |
| --- | --- |
| Equation | A+B/u+C*u |
| A (µm) | 4.0 ± 1.1 |
| B (mm^2^ms^-1^) | 0.8± 0.2 |
| C (ms) | 7.4± 0.7 |

Fig. S2: Purity determination of the collected fraction F1 (corresponding to E1) and F2 (corresponding to E2) from Chiralpak ZWIX(+) preparative LC separation (a middle fraction was removed to improve purity at expense of yield).

Fig. S3: The RP chromatograms of the collected fractions for the confirmation of the mass by HRMS using ESI negative mode. (a) EIC of *m/z* 197.022 of F1 (trace in black) and F2 (trace in red), (b) MS1 spectrum, (c) MS2 spectrum of the precursor ion [M-H]^-^ with *m/z* 197.022.

Fig. S4: Preparative separation of phosphonothrixin on a Chiralpak ZWIX(-) column. Overlay of 20 injections with Rs = 2.4.

Fig. S5: Purity determination of the collected fraction F1 (E1) and F2 (E2) from Chiralpak ZWIX(-) preparative LC separation.

Fig. S6: Reversal of elution order on a Chiralpak ZWIX(-) column compared to a Chiralpak ZWIX(+) column. The light blue chromatogram shows the separation of the racemic phosphonothrixin (PPX) on a Chiralpak ZWIX(-) column; the red chromatogram corresponds to F2 (E1) collected from a ZWIX(+) column and reinjected on a ZWIX(-) columns, and the black chromatogram corresponds to F1 (E2) collected from a ZWIX(+) columns and reinjected on a ZWIX(-) column.

Fig. S7: Overlay of CD spectra of first (E1) and second eluted enantiomer (E2) of phosphonothrixin on a) a Chiralpak ZWIX(+) column and b) a ZWIX(-) column.

Fig. S8: Experimental and population-averaged (50/50% and 60/40%), calculated CD spectra of *mono*- and *di*-anionic phosphonate species of (*R*)- and (*S*)-enantiomers of phosphonothrixin. Thin lines represent the spectra of *mono*- and *di*-anionic species separately. Dotted lines represent the resulting prorated, combined spectra of the respective enantiomer. E1 and E2 refer to the first (E1) and second (E2) eluted enantiomers on Chiralpak ZWIX(-).

**Synthesis of** **dibenzyl (2-hydroxy-2-(hydroxymethyl)-3-oxobutyl)phosphonate (DBP)**

In a Schlenk round bottom flask under N_2_-atmosphere was added 3.68 mL dibenzyl phosphite (4.37 g, 16.7 mmol, 5.00 equiv.) in 100 mL dry THF, and the mixture was cooled to -78 °C. Afterwards, a solution of 8.30 mL isopropyl magnesium chloride (2.00 M in THF, 1.71 g, 16.7 mmol, 5.00 equiv.) was added dropwise, and the resulting reaction mixture stirred for 15 min at -78 °C before adding a solution of 540 mg (2-(1,1-dimethoxyethyl)oxiran-2-yl)methanol (3.33 mmol, 1.00 equiv.) in 15.0 mL dry THF. The resulting solution was stirred for further 30 min at -78 °C and then another 1 h at rt. Afterwards, the reaction mixture was quenched with 40.0 mL of 1 M aq. HCl, and the resulting aq. layer was extracted three times with EtOAc. The combined organic layers were dried over Na_2_SO_3,_ and the solvent removed under reduced pressure.

The crude product was purified by flash column chromatography (SiO_2_, 50% to 100% ethyl acetate in n-hexane) yielding 960 mg DBP (2.53 mol, 76%) as a colorless oil that solidifies over time. The analytical data corresponds to literature [1].

**^1^H NMR** **(400 MHz, CDCl_3_ δ):** 7.42 – 7.28 (m, 10H, CH_Ar_), 5.07 – 4.84 (m, 4H, POCH_2_C_Ar_), 3.75 – 3.56 (m, 2H, HOCH_2_C_q_), 2.30 (dd, J = 18.4, 1.6 Hz, 2H, C_q_CH_2_P), 2.26 (s, 3H, CH_3_O).

**^13^C NMR** **(101 MHz, CDCl_3_ δ):** 210.86 (d, J = 7.1 Hz), 135.9, 135.8, 128.8, 128.3, 79.7 (d, J = 5.7 Hz), 68.0 – 67.9 (m), 67.7, 32.2, 30.8, 25.6.

**Synthesis of dibenzyl ((4-acetyl-2,2-dimethyl-1,3-dioxolan-4-yl)methyl)phosphonate (ABP)**

In a Schlenk round bottom flask under N_2_-atmosphere was added 100 mg dibenzyl (2-hydroxy-2-(hydroxymethyl)-3-oxobutyl)phosphonate (264 mmol, 1.00 equiv.), 66.0 mL 2,2-dimethoxy propane (56.1 mg, 529 mmol, 2.00 equiv.), 25.1 mg *p*-toluenesulfonic acid monohydrate (132 mmol, 0.50 equiv.) and 159 mg MgSO_4_ (1.32 mmol, 5.00 equiv.) to 25 mL dry acetone, and the reaction mixture was stirred at rt overnight. The reaction was subsequently quenched by addition of solid NaHCO_3_, and the reaction mixture filtered. Afterwards, the solvent was removed under reduced pressure, and the resulting crude product was purified by flash column chromatography (SiO_2_, 30% to 100% ethyl acetate in hexane) yielding 87.6 mg ABP (321 mmol, 79%) as a colorless oil.

**^1^H NMR (400 MHz, CDCl_3_ δ):** 7.37 – 7.28 (m, 10H, CHAr), 5.02 – 4.86 (m, 4H, OCH_2_CAr), 4.08 (dd, *J* = 9.3, 3.7 Hz, 1H, C_q_CH_2_O), 3.87 (dd, *J* = 9.3, 0.9 Hz, 1H, C_q_CH_2_O), 2.71 – 2.25 (m, 2H, C_q_CH_2_P), 2.28 (s, 3H, CH_3_CO), 1.44 (s, *J* = 0.8 Hz, 3H, CH_3_C_q_), 1.40 (s, 3H, CH_3_C_q_).

**^13^C NMR (101 MHz, CDCl_3_ δ):** 210.2, 136.4 (d, *J* = 6.5 Hz), 136.2 (d, *J* = 6.5 Hz), 128.7, 128.5, 128.2 (d, *J* = 7.2 Hz), 112.2, 85.7 (d, *J* = 3.8 Hz), 72.1 (d, *J* = 13.9 Hz), 67.6 (d, *J* = 6.3 Hz), 67.3 (d, *J* = 6.3 Hz), 35.4, 33.9, 26.7, 26.2, 26.1.

**^31^P NMR (162 MHz, CDCl_3_, reference 85% H_3_PO_4_ δ):** 27.0.

**IR (film, cm^−1^):** 1717 (m, CO), 1498 (w), 1456 (w), 1374 (m), 1354 (w), 1246 (s), 1216 (s), 1179 (w), 1153 (w), 993 (b), 922 (w), 882 (w), 838 (m), 734 (s), 697 (s), 595 (w), 523 (w), 460 (w), 405 (w).

**HR-ESI-TOFMS *m/z* [M+H]^+^:** 419.1632.

**Synthesis of** **(2-hydroxy-2-(hydroxymethyl)-3-oxobutyl)phosphonic acid (PPX)**

In a round bottom flask was added 100 mg dibenzyl (2-hydroxy-2-(hydroxymethyl)-3-oxobutyl)phosphonate (264 mmol, 1.00 equiv.) and 28.1 mg Pd/C (5 w%, 13.2 mmol, 0.05 equiv.) to 20 mL H_2_O. The resulting suspension was flushed with H_2_ and then kept under H_2_-atmosphere for 3 h at 60 °C with continuous stirring. Afterwards, the Pd/C catalyst was filtered off, and the aq. solution was frozen and then lyophilized under high vacuum to obtain 52.5 mg PPX (264 mmol, 99%) as a white solid. The analytical data corresponds to [1].

**^1^H NMR** **(400 MHz, D_2_O, δ):** 3.75 (dd, J = 76.7, 11.8 Hz, 2H, HOCH_2_C_q_), 2.39 – 2.05 (m, 5H, CH_3_CO, C_q_CH_2_P).

[1]	K. Nakamura, Y. Matsushima, Enantioselective total syntheses of (S)-phosphonothrixin and unexpected cyclic derivative (S)-cyclic phosphonothrixin via enzymatic resolution, Biosci. Biotechnol. Biochem. (2022) 1–10. https://doi.org/10.1093/bbb/zbac188.
